# Linking bacterial tetrabromopyrrole biosynthesis to coral metamorphosis

**DOI:** 10.1038/s43705-023-00309-6

**Published:** 2023-09-19

**Authors:** Amanda T. Alker, Morgan V. Farrell, Alyssa M. Demko, Trevor N. Purdy, Sanjoy Adak, Bradley S. Moore, Jennifer M. Sneed, Valerie J. Paul, Nicholas J. Shikuma

**Affiliations:** 1 Department of Biology and Viral Information Institute, San Diego State University, San Diego, CA 92182 USA; 2 Smithsonian Marine Station, Ft. Pierce, FL 34949 USA; 3 Center for Marine Biotechnology and Biomedicine, Scripps Institution of Oceanography, University of California, San Diego, La Jolla, CA 92093 USA

**Keywords:** Water microbiology, Bacterial genetics

## Abstract

An important factor dictating coral fitness is the quality of bacteria associated with corals and coral reefs. One way that bacteria benefit corals is by stimulating the larval to juvenile life cycle transition of settlement and metamorphosis. Tetrabromopyrrole (TBP) is a small molecule produced by bacteria that stimulates metamorphosis with and without attachment in a range of coral species. A standing debate remains, however, about whether TBP biosynthesis from live *Pseudoalteromonas* bacteria is the primary stimulant of coral metamorphosis. In this study, we create a *Pseudoalteromonas* sp. PS5 mutant lacking the TBP brominase gene, *bmp2*. Using this mutant, we confirm that the *bmp2* gene is critical for TBP biosynthesis in *Pseudoalteromonas* sp. PS5. Mutation of this gene ablates the bacterium’s ability in live cultures to stimulate the metamorphosis of the stony coral *Porites astreoides*. We further demonstrate that expression of TBP biosynthesis genes is strongest in stationary and biofilm modes of growth, where *Pseudoalteromonas* sp. PS5 might exist within surface-attached biofilms on the sea floor. Finally, we create a modular transposon plasmid for genomic integration and fluorescent labeling of *Pseudoalteromonas* sp. PS5 cells. Our results functionally link a TBP biosynthesis gene from live bacteria to a morphogenic effect in corals. The genetic techniques established here provide new tools to explore coral-bacteria interactions and could help to inform future decisions about utilizing marine bacteria or their products for coral restoration.

Some marine bacteria stimulate the early life-cycle transition from larval to juvenile phases in corals (i.e., metamorphosis) [1–3]. These bacteria could be used to promote coral larval recruitment in the wild, cultivate corals for reseeding degraded reefs or the aquarium trade, or help test basic science questions in the laboratory. Single-species biofilms of *Pseudoalteromonas* bacteria, such as *Pseudoalteromonas* sp. strain PS5, have been shown to stimulate coral metamorphosis [2, 4, 5]. Moreover, the compound tetrabromopyrrole (TBP), extracted from *Pseudoalteromonas* bacteria or chemically synthesized, robustly promotes the metamorphosis of diverse coral species with and without attachment [2, 5–7] (Fig. 1A). However, an open question remains about whether live *Pseudoalteromonas* bacteria stimulate coral larval metamorphosis solely by producing TBP, or whether these *Pseudoalteromonas* strains produce yet unknown products that confer part or all of the stimulatory activity [6, 8].

**Fig. 1:**
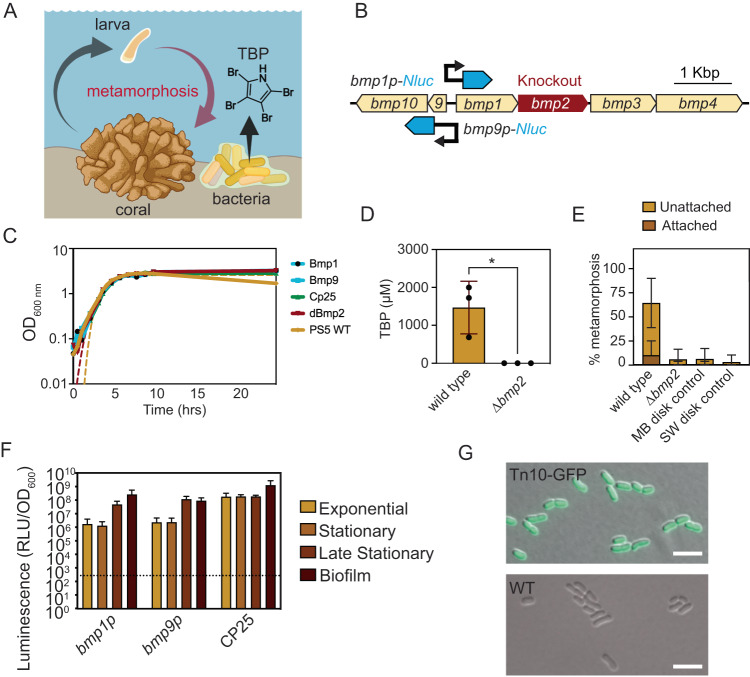
TBP biosynthesis in *Pseudoalteromonas* sp. PS5 induces metamorphosis of the stony coral *Porites astreoides*. **A** A model of TBP biosynthesis in *Pseudoalteromonas* sp. PS5 and its ability to induce coral metamorphosis. **B** Synteny of the *bmp* gene cluster in *Pseudoalteromonas* sp. PS5 (Genbank accession KR011923) created with EasyFig (V1.4.4) [23]. The 5’UTR for both *bmp1* and *bmp9* were cloned and fused to a *Nanoluc* (*Nluc*) reporter shown in blue. The *bmp2* brominase is highlighted in red. **C** Growth curve of *Pseudoalteromonas* sp. PS5 wild type, ∆*bmp2* and strains expressing plasmids *bmp1-NLuc*, *bmp9-NLuc*, and CP25*-gfp*. Optical density (OD) measurements were taken at 600 nm wavelength and graphed on a Log_10_ scale. Dotted lines correspond to the Gompertz non-linear regression fit for wild type and ∆*bmp2*. Plotted is the average of two biological replicates. **D** LC-MS/MS of *Pseudoalteromonas* sp. PS5 wild type and *bmp2* knockout quantifying tetrabromopyrrole production (*N* = 3 extractions per treatment extracted from replicate 5 mL liquid cultures, one-tailed Mann–Whitney test, *P* = 0.05). Error bars show standard deviations. **E** Metamorphosis biofilm assays (%) with *Porites astreoides* larvae (10 larvae/ dish) in response to *Pseudoalteromonas* sp. PS5 wild type and ∆*bmp2* strains. Controls include disks incubated in sterile Marine Broth (MB) media or filtered seawater (SW). *N* = 25 total dishes per treatment, including 3 experiments with either 5 or 10 replicates spanning two collection years. Combined morphogenesis significance is shown (Dunn’s multiple comparison of wild type vs *∆bmp2*, Adjusted *P* < 0.0001). **F** Luciferase assays of *bmp1* and *bmp9* promoter activity under different modes of growth reported in relative luminescence units normalized by the optical density (RLU/OD_600_) and plotted on the Log_10_ scale. The 5’UTR of *bmp1* and *bmp9* were compared against the negative control background (*Pseudoalteromonas* sp. PS5 cells expressing a non-luminescent plasmid) as represented by the dotted line (Y = 60,422 RLU/OD_600_). Error bars show standard deviation of the mean. *N* = 4 biological replicates. **G** Strong and uniform expression of the genomic GFP tag in the *Pseudoalteromonas* transconjugants observed with fluorescence microscopy (top). The bottom panel is the wild type *Pseudoalteromonas* sp. PS5 for comparison. Scale bar is 5 µm.

In *Pseudoalteromonas* sp. PS5 and phylogenetically related strains, TBP biosynthesis from L-proline is performed by three enzymes and a carrier protein encoded by the brominated marine pyrroles/phenols (*bmp*) gene cluster [9], specifically genes *bmp1-4* [10] (Fig. 1B). We thus sought to create a TBP deletion mutant in *Pseudoalteromonas* sp. PS5 to query coral larval metamorphosis by genetically inactivating the *bmp2* gene, which codes for the brominase that installs all four bromine atoms in TBP [10]. We determined that *Pseudoalteromonas* sp. PS5 is amenable to genetic manipulation by conjugation using a broad-host-range *gfp* reporter plasmid from the Marine Modification Kit (MMK) plasmid system [11]. We generated a *Pseudoalteromonas* sp. PS5 mutant with an in-frame deletion of the *bmp2* gene (∆*bmp2*) following previously established methods for double-homologous recombination [12] (Fig. 1B). The modified strains exhibited a similar growth rate to wild type; however, the plasmid containing and *bmp2* mutant strains persisted longer in stationary phase (Fig. 1C). The mutation of *bmp2*, as anticipated, resulted in the complete loss of TBP production in the ∆*bmp2* strain in stark contrast to the wild-type culture that readily produces TBP (1468 ± 401 µM TBP, *P* = 0.05, one-tailed Mann–Whitney test) (Fig. 1D). These results confirm that the *bmp2* gene is required for TBP production in *Pseudoalteromonas* sp. PS5 under the conditions tested.

We next tested whether bacteria lacking the *bmp2* gene were able to stimulate the metamorphosis of the Caribbean coral, *Porites astreoides*, which has been shown previously to undergo metamorphosis in response to *Pseudoalteromonas* sp. PS5 and TBP [2]. When exposed to biofilms of wild type *Pseudoalteromonas* sp. PS5, we observed the metamorphosis of coral larvae consistent with previous findings [2, 4–6], both attached to the substrate (11.6% ± 7.2, Adjusted *P* = 0.0002, Dunn’s multiple comparisons test) and unattached (55% ± 13.1, Adjusted *P* < 0.0001, Dunn’s multiple comparisons test) (Fig. 1E, Supplementary Table S1). In contrast, total morphogenesis of coral larvae was reduced from 63.5% in wild type biofilm treatments to 3.5% in the ∆*bmp2* biofilm treatment (Adjusted *P* < 0.0001, Dunn’s multiple comparisons test) (Fig. 1E, Supplementary Table S1). We observed attached (0.6% ± 2.9) and unattached (5.1% ± 10.4) metamorphosis in the ∆*bmp2* treatment at rates comparable to the background in the media disk and the seawater disk (placed directly in the Petri dishes with filtered seawater) controls. Our results demonstrate that the effect of *Pseudoalteromonas* sp. PS5 on coral metamorphosis is primarily due to the production of TBP.

We then questioned whether different growth conditions affect the expression of the *bmp* genes in *Pseudoalteromonas* sp. PS5. To this end, we cloned the *bmp1* and *bmp9* promoters, fused them with a *NanoLuciferase* (*NLuc*) reporter gene, and conjugated the resulting plasmids into *Pseudoalteromonas* sp. PS5 (Fig. 1B) [11]. Luminescence was measured during each growth phase with the highest activity measured in late stationary and biofilm phases (Fig. 1F). The *bmp1* promoter displayed a 203-fold increase in expression between early stationary and biofilm (Fig. 1F). The *bmp9* promoter followed similar expression profiles, suggesting that the gene cluster may be co-regulated. We also tested broad-host-range promoters, which displayed at least a 457-fold activity range compared to assay background across all tested conditions (Supplementary Fig. S1). Our results suggest that the expression of TBP biosynthesis genes is strongest when bacteria exist in a slow-growth state when *Pseudoalteromonas* sp. PS5 might occur within surface-attached biofilms on the sea floor.

*Pseudoalteromonas* species are known to associate with marine eukaryotes and produce interesting antimicrobial metabolites [13], yet the study of these host-microbe and microbe-microbe interactions remains challenging due to the limited genetic tools for their manipulation. We therefore developed an integrative Tn10 transposon for use in *Pseudoalteromonas* sp. PS5, which is compatible with existing modular genetic toolkit parts [11, 14, 15] (Supplementary Fig. S2). With the Tn10 transposon, we generated *Pseudoalteromonas* sp. PS5 with *gfp*-tags integrated into the genome (Fig. 1G, Supplementary Fig. S2), which were confirmed by whole genome sequencing to identify genomic insertion loci (Supplementary Table S2).

In this work, the genetic engineering of a marine microbe enabled us to test a standing question about the role of TBP in coral metamorphosis. Our results represent the first characterization of a gene in a marine bacterium conveying a morphogenic effect in corals. Previous studies suggest that *Pseudoalteromonas* species may not be present in ecologically relevant concentrations that would stimulate coral metamorphosis [6]. However, TBP could be used as a molecule to elucidate mechanisms of coral morphogenesis. The strains and methodological advancements developed in this study could be helpful for dissecting how TBP stimulates metamorphosis with and without attachment in corals [16] and more broadly for studying TBP’s effects on eukaryotic cell physiology [17–20]. Furthermore, these approaches could be applied to inform responsible use of coral probiotic strain candidates encoding the *bmp* gene cluster[21]. Beyond TBP, our results demonstrate how bacterial genetics can help characterize genes and gene products from bacteria in the context of non-model marine microbial interactions, providing new techniques to interrogate the microbial ecology of *Pseudoalteromonas* spp. We hope this work will represent a step towards elucidating function in bacteria-coral interactions and will inform the use of bacteria for coral reef restoration [22].

### Supplementary information

Supplemental Material

## Data Availability

Key plasmids generated for this study are available through Addgene (https://www.addgene.org/Nicholas_Shikuma/).
